# Agr typing of *Staphylococcus aureus* species isolated from clinical samples in training hospitals of Isfahan and Shahrekord

**DOI:** 10.1186/s13104-019-4396-8

**Published:** 2019-06-27

**Authors:** Saeid Javdan, Tahmine Narimani, Milad Shahini Shams Abadi, Abolfazl Gholipour

**Affiliations:** 1 Cellular and Molecular Research Center, Basic Health Sciences Institute, Shahrekord University of Medical Sciences, Shahrekord, Iran; 2 Department of Microbiology, Isfahan University of Medical Sciences, Isfahan, Iran; 3 Department of Bacteriology & Virology, School of Medicine, Shiraz University of Medical Sciences, Shiraz, Iran; 4 Department of Microbiology and Immunology, Cellular and Molecular Research Center, Shahrekord University of Medical Sciences, Shahrekord, Iran

**Keywords:** *Staphylococcus aureus*, Agr type, mecA gene, Antibiotic resistance, Methicillin

## Abstract

**Objective:**

As an opportunistic pathogen, *Staphylococcus aureus* is associated with serious nosocomial infections and growing antimicrobial resistance against beta-lactams among *S. aureus* strains has become a global challenge. The current study was designed to investigate the presence of *agr* genes among *S. aureus* strains recovered from clinical samples in university hospitals of Isfahan and Shahrekord.

**Results:**

A total of 150 *S. aureus* isolates were screened by Disk diffusion method (DDM) and conventional PCR. The minimum (17.3%) and maximum (46%) antibiotic resistance rates were found in vancomycin and cefoxitin, respectively. The majority of our isolates were classified as *agr* type I followed by type II, type IV, and type III. The statistical analysis showed a significant correlation between *agr* type I and antibiotic resistance against cefoxitin and erythromycin (p = 0.04 and p = 0.03, respectively). Based on our findings, the *agr* typing could be considered an effective approach for molecular tracking of *S. aureus* infections.

## Introduction

As a part of microflora of skin and mucous membranes of healthy individuals, *Staphylococcus aureus* is also an opportunistic pathogen and associated with hospital acquired infections such as septicemia, pneumonia, septic arthritis, osteomyelitis, toxic shock syndrome after surgery, folliculitis, endocarditis, and urinary tract infections (UTIs) [1, 2]. Antibiotic resistance by affecting more than two million people annually is one of the biggest global challenges. The increasing antimicrobial resistance among *S. aureus* species against beta-lactam antibiotics has led to serious problems with the treatment of their related infections. Despite considerable efforts in controlling antibiotic resistance, methicillin-resistance *S. aureus* (MRSA) is raising worldwide, in addition geographical and local variations influence its dynamic and crisis [1, 3]. The methicillin resistance development in *S. aureus* is related to the several Staphylococcal Cassette chromosome *mec* elements (SCCmec) encoding *mecA* gene for a penicillin binding protein (PBP2a) [4]. MRSA strains are usually multi-drug resistant (MDR) and show resistancy to other antibiotics such tetracyclines, aminoglycosides, lincosamides etc. [1, 5, 6]. Rapid and precise typing of *S. aureus* is really crucial to transmission identification of this pathogen. In this regard, Pulsed-Field gel electrophoresis and *spa* typing (Staphylococcal protein A) are common typing methods. The spa gene is one of the most distinctive factors related to this organism, and various patterns of it have been identified by several studies [7]. One of the major regulatory and control factors in the virulence gene expression of *S. aureus* is the accessory gene regulatory (*agr*) system. Indeed, *agr* operon including *agrA*, *agrB*, *agrC*, and *agrD* genes regulate over 70 genes in *S. aureus* 23 of which control its pathogenicity and invasive infections [8]. Moreover, *S. aureus* can be stratified into 4 different groups (agr I, agr II, agr III, and agr IV) according to the sequences of *agrC* (auto inducing peptide) and *agrD* (cyclic AIP) genes. It is stated that *agr* types are different in their properties and prevalence in various geographical areas thus, identification of predominant types in each region may well be functional [9].

Given to the critical roles of *agr* genes, the current study was designed to detect and identify the *agr* groups of *S. aureus* strains isolated from clinical samples in training hospitals of Isfahan and Shahrekord cities.

## Main text

### Materials and methods

#### Samples and bacterial isolates

This cross sectional study was conducted in microbiology department of Shahrekord University of Medical Sciences. During May to November 2017, a total of 150 isolates of *S. aureus* were collected from clinical samples (wound, blood, urine, tissue etc.) of patients attending university hospitals in Isfahan (Alzahra and Kashani) and Shahrekord (Kashani and Hajar).

#### Characterization assays

The isolates were identified using Gram staining, catalase test, slide or tube coagulase test, DNase test, and growth on Mannitol Salt Agar (MSA) as a differential growth medium [10]*.*

#### Antibiogram testing

Disk diffusion method (DDM) as described by CLSI 2016 guideline [11] was performed for following antibiotics: erythromycin (15 mg), tetracycline (30 mg), vancomycin (30 mg), gentamicin (10 mg), rifampin (5 mg), cefoxitin (15 mg), trimethoprim (5 mg), rifampicin (5 mg). In addition, all isolates were subjected to cefoxitin disc diffusion test to identify the methicillin sensitive *S. aureus* (MSSA) and MRSA.

#### DNA extraction

The nucleic acids of *S. aureus* isolates were extracted by phenol chloroform method followed by RNase treatment [12]. The purity of extraction was assessed using the A260/280 ratio and agarose gel electrophoresis.

#### PCR amplification of the mecA gene

molecular detection of *mecA* gene was carried out according to the following condition: initial denaturation at 95 °C for 3 min followed by 33 cycles of denaturation at 94 °C for 1 min, annealing at 53 °C for 30 s, and extension at 72 °C for 1 min and final extension step at 72 °C for 6 min.

#### PCR detection of agr genes

PCR assay for amplification of *agr* genes was set as follows: hot start at 95 °C/6 min, 32 cycles of 94 °C/45 s, 60 °C/1 min, 72 °C/70 s and a final extension step of 72 °C/8 min. All reactions performed in duplicate and along with the negative control (water) and positive (previously known positive-PCR products) control. The final products were detected by electrophoresis on 1% agarose gel containing DNA safe stain (Sinagene, Iran) and the sizes of the PCR products were estimated by the migration pattern of a 100-bp DNA ladder (Sinagene, Iran).

#### Statistical analysis

Statistical analysis was performed using SPSS version 22. The chi-square test was used to calculate statistical significance (p < 0.05).

### Results

#### Study population

150 *S. aureus* isolates were collected from patients attending training hospitals in Isfahan (110 isolates from Alzahra hospital) and Shahrekord (25 cases from Kashani hospital and15 isolates from Hajar hospital). The mean age of the participants was 47.6 years (SD: 21.5) and male/female ratio was 90/60. However, there was not any significant difference in sex and age of patients with *S. aureus* infection. *S. aureus* isolates were obtained from several clinical samples and different hospital wards.

#### Antibiotic susceptibility

According to our results the lowest (17.3%) and the highest (46%) antibiotic resistance rates were found in vancomycin and cefoxitin, respectively. In addition, MRSA strains were verified by PCR amplification of mecA gene. The antibiotic resistance distribution among different *agr* groups is shown in Table 1. The results of this study showed a significant correlation between *agr* type and antibiotic resistance against cefoxitin and erythromycin (p = 0.04 and p = 0.03, respectively).

**Table 1:** The antibiotic resistance profiles among 4 different *agr* types

AGR type	FOX		E			T			VA		RP			TM			GM		
	S (%)	R (%)	S (%)	I (%)	R (%)	S (%)	I (%)	R (%)	S (%)	R (%)	S (%)	I (%)	R (%)	S (%)	I (%)	R (%)	S (%)	I (%)	R (%)
I	37 (45.1%)	45 (54.9%)	38 (46.3%)	21 (25.6%)	23 (28)	47 (57.3%)	7 (8.5%)	28 (34.1%)	70 (85.4%)	12 (14.6%)	49 (59.8%)	3 (3.7%)	30 (36.6%)	52 (63.4%)	6 (7.3%)	24 (29.3%)	58 (70.7%)	2 (2.4%)	22 (26.8%)
II	22 (59.5%)	15 (40.5%)	12 (32.4%)	5 (13.5%)	20 (54.1%)	20 (54.1%)	1 (2.7%)	16 (43.2%)	28 (75.7%)	9 (24.3%)	27 (73.0%)	2 (5.4%)	8 (21.6%)	18 (48.6%)	5 (13.5%)	14 (37.8%)	23 (62.2%)	0	14 (37.8%)
III	7 (70%)	3 (30%)	3 (30%)	1 (10%)	6 (60%)	7 (70%)	0	30 (30%)	8 (80%)	2 (20%)	8 (80%)	1 (10%)	1 (10%)	4 (40%)	1 (10%)	5 (50%)	7 (70%)	1 (10%)	2 (2%)
IV	15 (71.4%)	6 (28.6%)	6 (28.6%)	7 (33.3%)	8 (38.1%)	10 (47.6%)	1 (4.8%)	10 (47.6%)	18 (85.7%)	3 (14.3%)	14 (66.7%)	1 (4.8%)	6 (28.6%)	14 (66.7%)	1 (4.8%)	6 (28.6%)	13 (61.9%)	0	8 (38.1%)
Total	81 (54%)	69 (46%)	59 (39.3%)	34 (22.7%)	57 (38%)	84 (56%)	9 (6%)	57 (38%)	124 (82.7%)	26 (17.3%%)	98 (65.3%)	7 (4.7%)	45 (30%)	88 (58.7%)	13 (8.7%)	49 (32.7%)	101 (67.3%)	3 (2%)	46 (30.7%)

#### agr typing

**Fig. 1:**
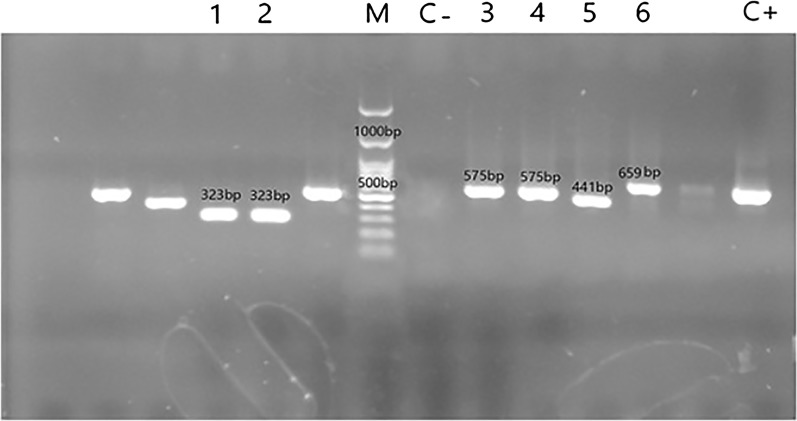
Lanes 1 and 2: agr type III, lanes 3 and 4: agr type II, lane 5: agr type I, lane 6: agr type IV, M: 100 bp DNA ladder, C− and C+: negative and positive controls

Molecular detection of 150 *S. aureus* isolates has indicated that *agr* type I was the predominant one (82/150) followed by type II (37/150), type IV (21/150), and type III (10/150) (Fig. 1). Table 2 is shown the frequency distribution of different *agr* types among different clinical samples.

**Table 2:** Frequency distribution of *agr* types in different clinical samples

Sample	*agr* type				Total
	I	II	III	IV	
Tracheal aspirate					
No	13	6	2	2	23
%	56.5%	26.1%	8.7%	8.7%	100%
Wound					
No	30	11	4	5	50
%	60.0%	22.0%	8.0%	10.0%	100%
Tissue					
No	3	2	0	0	5
%	60.0%	40.0%	0.0%	0.0%	100%
Abscess					
No	5	5	1	1	12
%	41.7%	41.7%	8.3%	8.3%	100%
Conjunctival swab					
No	0	1	0	1	2
%	0.0%	50.0%	0.0%	50.0%	100%
Blood culture					
No	1	2	0	0	3
%	33.3%	66.7%	0.0%	0.0%	100%
Sputum					
No	2	0	0	2	4
%	50.0%	0.0%	0.0%	50.0%	100%
Nasal aspirate					
No	2	1	1	0	4
%	50.0%	25.0%	25.0%	0.0%	
Synovial fluid					
No	0	0	0	3	3
%	0.0%	0.0%	0.0%	100%	100%
Discharge					
No	3	3	1	0	7
%	42.9%	42.9%	14.3%	0.0%	100%
Bactace					
No	7	1	0	2	10
%	70.0%	10.0%	0.0%	20.0%	100%
Blood					
No	4	3	0	1	8
%	50.0%	37.5%	0.0%	12.5%	100%
Peritoneal fluid					
No	1	0	1	0	2
%	50.0%	0.0%	50.0%	0.0%	100%
Urine culture					
No	9	2	0	3	14
%	64.3%	14.3%	0.0%	21.4%	100%
Pharyngeal swab					
No	2	0	0	1	3
%	66.7%	0.0%	0.0%	33.3%	100%
Total					
No	82	37	10	21	150
%	54.7%	24.7%	6.7%	14.0%	100%

### Discussion

There is a dramatic increase in *S. aureus* infections, both with community-associated and hospital-acquired types, and development of antibiotic-resistant species, especially MRSA and vancomycin-resistant strains, is the major cause of the infections and further treatment complications [13]. Identification and typing of the isolates may imply a common source of infection; therefore, accurate analysis of these patterns can help to break the chain of transmission. Accordingly, the present study was designed to identification of *agr* types among *S. aureus* isolates and possible association of these pathogens with some phenotypic characteristics such as antibiotic resistance and pathogenesis.

Dufour et al. [14] used *agr* typing method for the first time to stratify *S. aureus* isolates and affirmed that these bacteria can be divided into four groups I, II, III, IV by this system. Ever since, many researches have been applied the *agr* typing approach and in several studies such as those by Lee et al. and Shopsin et al. [15, 16], the *agr* group I was the most dominant *S. aureus* type. Our findings indicated that *agr* type I was the most predominant type among *S. aureus* isolated from Isfahan and Shahrekord cities. Similarly, in several previous studies such as those by Cheraghi, Bibalan, Peerayeh, Khoramrooz, Mohsenzadeh, and Goudarzi *agr* type I has been reported as the most dominant isolate of *S. aureus* in different regions of Iran [17–22]. It is declared that certain *agr* groups of *S. aureus* are involved in some particular disease and infections, for example *agr* type I isolates are associated with bacteremia and invasive infections [21]. In the present study, wound and tracheal aspirates, were sequentially the most frequent clinical samples and as it is summarized in Table 2, the *agr* group I was the major *agr* type among these sample. However, we couldn't find any significant difference or correlation between *agr* types and certain clinical specimen.

In the current study, the antimicrobial susceptibility testing revealed that the highest antibiotic resistance rate was against cefoxitin (46%) followed by erythromycin and tetracycline (both 38%). Several studies that have reported erythromycin and tetracycline as the antimicrobial agents with lowest susceptibility among *S. aureusagr* group I isolates [20, 23, 24]. As it is summarized in Table 1, the agr types III and I showed the maximum and minimum resistance rates against tetracycline, respectively. In the present study, agr type I isolates had the highest sensitivity to vancomycin; however, the smallest resistance rate against this agent was related to agr type IV (Table 1). The greatest susceptibility and resistance to rifampin were found among agr types IV and I of *S. aureus* strains, respectively (Table 1). As it could be seen in Table 1, agr types IV and III have shown the highest and the lowest susceptibility to trimethoprim, respectively. The maximum percentage of gentamicin susceptibility was related to agr type I, while type III isolates had the highest resistance against this antimicrobial agent (Table 1). We found a significant correlation between *agr* type and antibiotic resistance against cefoxitin and erythromycin (p = 0.04 and p = 0.03, respectively). Indeed, in this report, the *agr* types never implied the sensitivity or resistance to antibiotics, but in the case of cefoxitin and erythromycin the *agr* group I isolates showed the highest resistance against these agents.

The majority of *S. aureus* isolates in this study were classified as *agr* group I and our results suggest a probable correlation between this type and antibiotic resistance to cefoxitin and erythromycin. Here we can conclude that *agr* typing is a suitable and effective approach for molecular tracking of *S. aureus* infection.

## Limitations

The lack of investigation on others typing methods in *S. aureus* isolates can be mentioned as one of the main limitations of the present study.

## Data Availability

All relevant data are included in the manuscript.

## Abbreviations

MRSA: methicillin-resistance *Staphylococcus aureus*
MDR: multi-drug resistant
SCCmec: Staphylococcal Cassette chromosome mec elements
PCR: polymerase chain reaction
